# Multitechnological integration advances musculoskeletal regeneration: synergistic progress of organoids, 3D/4D bioprinting, single-cell omics and artificial intelligence

**DOI:** 10.3389/fbioe.2026.1824644

**Published:** 2026-05-29

**Authors:** Zhichao Liu, HaiYan Fan, TianMin Zhai, ZhongPin Ma, Yun Yang

**Affiliations:** 1 Graduate School, Inner Mongolia Medical University, Hohhot, Inner Mongolia, China; 2 Imaging Center, Affiliated Hospital of Inner Mongolia Medical University, Hohhot, Inner Mongolia, China; 3 Center for Joint Surgery, The Second Affiliated Hospital of Inner Mongolia Medical University, Hohhot, Inner Mongolia, China; 4 Center for Joint Surgery, The Second Affiliated Hospital of Inner Mongolia Medical University, Hohhot, Inner Mongolia, China

**Keywords:** 3D bioprinting, 4D bioprinting, artificial intelligence, multitechnological integration, musculoskeletal regeneration, organoids, single-cell omics

## Abstract

Regenerative repair of injuries and degenerative diseases in the musculoskeletal system remains a paramount clinical challenge, as conventional single-modal technologies fail to recapitulate the intricate structural and functional complexity of native musculoskeletal tissues. In recent years, the rapid evolution of cutting-edge technologies-including organoids, 3D/4D bioprinting, single-cell omics and artificial intelligence (AI)-has unlocked novel opportunities for addressing this longstanding issue. This review aims to systematically elucidate how the integration of these four core technologies drives progress in musculoskeletal regeneration research. By exploring the intrinsic methodological details of each technology and their underlying synergistic mechanisms, we summarize the latest advances in multitechnological integration for fabricating highly biomimetic *in vitro* models, enabling precise and dynamic fabrication of tissue-engineered constructs, deciphering cellular heterogeneity during tissue development and repair at high resolution, and optimizing data-driven personalized regenerative strategies. We further explicitly distinguish the *in vitro* and *in vivo* applications of each technology with representative experimental evidence and translational implications, and provide comprehensive tabular summaries of state-of-the-art research over the past 5 years. Furthermore, we prospect the future development directions and critical challenges facing this interdisciplinary field.

## 1 Introduction

Musculoskeletal tissues, comprising bone, cartilage, skeletal muscle and tendon, are indispensable for human motor function and structural support. However, their regenerative repair post-injury continues to pose a formidable challenge in clinical practice (Peniche et al., 2026). The regeneration of these tissues is governed by intricate molecular signaling cascades that orchestrate the sequential inflammatory, reparative and remodeling phases (Peniche et al., 2025). Deciphering these molecular cues is pivotal for the rational design of targeted therapeutic strategies (Peniche et al., 2025). Within the musculoskeletal system, bone, tendon and muscle form highly integrated multi-tissue units (e.g., the rotator cuff complex), whose injuries not only inflict severe morbidity on patients but also impose a substantial socioeconomic burden on global healthcare systems (Zhang et al., 2022a). Despite remarkable advancements in tissue engineering and regenerative medicine over the past decades—where functional tissue substitutes are engineered by combining biomaterials, seed cells and bioactive signaling factors—conventional approaches still struggle to accurately replicate the hierarchical architectures and dynamic microenvironments of native musculoskeletal tissues (Wei and Dai, 2021). For instance, osteochondral defect repair remains extremely challenging due to the spatial heterogeneity of such defects in composition, structure and biomechanical function (Wei and Dai, 2021). Similarly, the tendon-bone interface, a unique gradient structure bridging hard and soft tissues, presents great hurdles for effective healing and functional regeneration after injury (Chen et al., 2024a; Yin et al., 2026).

In recent years, the rapid development of a suite of revolutionary technologies has offered new avenues for overcoming these challenges. Organoid technology enables the generation of highly biomimetic three-dimensional *in vitro* microtissue models that recapitulate the structural and functional features of native tissues (Hsiung et al., 2025). To date, organoids of the liver, heart, skin and other organs have been widely applied in disease modeling, high-throughput drug screening and regenerative medicine research (Panwar et al., 2021; Ergir et al., 2022; Moradikhah et al., 2024). 3D bioprinting technology achieves precise spatial assembly of cells and biomaterials, facilitating the fabrication of tissue substitutes with complex anatomical morphologies (Chae and Cho, 2022; Liu et al., 2023). Going a step further, 4D bioprinting introduces a temporal dimension into tissue fabrication: printed constructs undergo preprogrammed morphological and functional evolution in response to exogenous or endogenous stimuli, thus better matching the dynamically changing biological microenvironment during tissue regeneration (Liu et al., 2026). Single-cell omics technologies—including single-cell RNA sequencing (scRNA-seq), single-cell epigenomics and single-cell proteomics—have unraveled cellular heterogeneity and cell fate transition trajectories during regeneration at the molecular level with unprecedented resolution (Negroni et al., 2022; Seeler et al., 2024; Zhou et al., 2025). For example, single-cell studies in skeletal muscle regeneration have delineated the heterogeneity of stromal cells (e.g., fibro-adipogenic progenitors) and the critical role of their “virtual” secretome in intercellular communication (Negroni et al., 2022). AI, on the other hand, empowers the analysis of massive multi-omics datasets, predictive modeling and the optimization of biomanufacturing processes (Wei et al., 2026a). AI and machine learning algorithms are increasingly being used to predict biomaterial properties, optimize 3D bioprinting parameters, analyze high-content imaging data, and even construct digital twin models to accelerate biomanufacturing and drug development pipelines (Maharjan et al., 2025; Almomani et al., 2026; Liu, 2025).

Notably, these four technologies are not isolated; their in-depth integration is fostering a novel research paradigm for musculoskeletal regeneration. The closed-loop workflow of model construction (organoids/bioprinting) → mechanism deciphering (single-cell/multi-omics) → precise fabrication (AI-optimized bioprinting) → intelligent iteration (AI-driven design and analysis) has drastically accelerated the translation from basic research to clinical applications (Hsiung et al., 2025; Lou et al., 2025). For example, combining organoids with 3D bioprinting yields tissue models with enhanced physiological relevance and structural controllability (Mierke, 2024; Liu et al., 2025a); AI can integrate multi-omics data to guide the optimization of bioink formulations and bioprinting strategies, enabling personalized tissue engineering (Wei et al., 2026a). Despite persistent challenges such as vascularization, long-term functional maintenance and clinical translation, multitechnological integration offers unprecedented opportunities for developing next-generation regenerative therapies and restoring the full physiological function of damaged musculoskeletal tissues (Şeker et al., 2024; Calin et al., 2025). In this review, we systematically elaborate on the synergistic advances of these four key technologies, and deeply analyze their integration strategies, representative applications in musculoskeletal regeneration and the critical challenges that need to be addressed. In this review, we not only elaborate the synergistic advances of these four key technologies, but also dissect their methodological principles, *in vitro*/*in vivo* application spectrum and latest research progress with detailed experimental data and standardized summaries, to provide a rigorous and practical reference for the translational research of musculoskeletal regeneration.

## 2 Applications of organoid technology in musculoskeletal regeneration modeling

### 2.1 Construction and biological characteristics of musculoskeletal organoids

Musculoskeletal organoids are typically engineered from pluripotent stem cells or tissue-specific progenitor cells via self-organization or bioengineering-guided culture, yielding miniature *in vitro* tissues that recapitulate the complex architectures of bone, cartilage, skeletal muscle or the tendon-bone interface (Chen et al., 2025). Different musculoskeletal organoid subtypes are constructed with tissue-specific cell sources, culture conditions and scaffold strategies (Table 1). For bone organoids, the primary cell sources include human induced pluripotent stem cells (hiPSCs), bone marrow mesenchymal stem cells (BMSCs) and periosteal progenitor cells; the culture system requires osteogenic induction medium supplemented with β-glycerophosphate (5–10 mM), ascorbic acid (50–100 μg/mL) and dexamethasone (10–100 nM), and 3D culture is commonly achieved via ultra-low attachment plates for self-aggregation or biomaterial scaffolds such as gelatin methacrylamide (GelMA) and hydroxyapatite (HA) (Huang et al., 2023; Holliday, 2023). Cartilage organoids are mainly derived from chondrogenic progenitor cells and hiPSCs, with chondrogenic induction medium containing transforming growth factor-β3 (TGF-β3, 10 ng/mL) and bone morphogenetic protein-2 (BMP-2, 50 ng/mL), and are typically cultured in hanging drops or alginate hydrogel microspheres to maintain chondrocyte phenotype and avoid hypertrophy (Chen et al., 2025; Lee et al., 2024). Skeletal muscle organoids rely on human primary myoblasts, satellite cells or hiPSC-derived myogenic progenitors, with culture conditions including 2% horse serum and myogenic differentiation factors such as myogenic differentiation 1 (MyoD), and scaffold-free self-assembly or Matrigel-based 3D culture to form contractile myotubes (Jalal et al., 2021; Maffioletti et al., 2022). Tendon/ligament organoids use dermal fibroblasts or tendon stem/progenitor cells (TSPCs), with a three-step culture protocol (expansion→stimulation→maturation) involving platelet-derived growth factor-BB (PDGF-BB) and TGF-β1, and self-assembled cell sheets to form rod-like constructs with layered ECM deposition (Graça et al., 2024; Fang et al., 2025).

**TABLE 1:** Latest research on musculoskeletal organoids (2020–2025).

Research	Model	Technology	Cell type	Material	Key findings	Year	Ref.
Bone organoid for osteogenesis imperfecta modeling	Human iPSC-derived bone organoid	Scaffold-free self-assembly	hiPSCs, BMSCs	β-glycerophosphate, ascorbic acid, dexamethasone	Bone organoids exhibit impaired mineralization and abnormal collagen deposition; denosumab restores mineralization by 40%	2023	Holliday, (2023)
Cartilage organoid for OA drug screening	Human OA cartilage organoid	Alginate hydrogel encapsulation	Chondrogenic progenitor cells	TGF-β3, BMP-2, alginate	SM04690 inhibits chondrocyte apoptosis and reduces MMP-13 expression by 50%	2024	Lee et al. (2024)
Skeletal muscle organoid for DMD therapy validation	Human DMD muscle organoid	Matrigel-based 3D culture	hiPSC-derived myogenic progenitors	MyoD, 2% horse serum	Exon-skipping therapy restores dystrophin expression in 30% of myotubes	2022	Maffioletti et al. (2022)
Tendon organoid for Achilles tendon repair	Rat tendon organoid	Self-assembled cell sheets	TSPCs, dermal fibroblasts	PDGF-BB, TGF-β1	Tendon organoids form mature tendon tissue with parallel collagen fibers	2025	Wang et al. (2025)
Patient-specific bone organoid for personalized regeneration	Human patient-derived bone organoid	HA/GelMA composite scaffold	Patient-derived iPSCs	HA, GelMA, osteogenic induction medium	Autologous bone organoid implantation achieves 80% calvarial defect regeneration	2024	Zhang et al. (2024)

**FIGURE 1:**
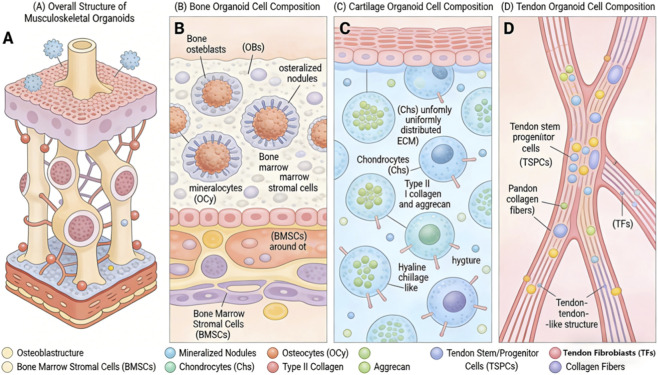
Schematic diagram of musculoskeletal organoid structure and cell composition. The figure illustrates the structural and cellular characteristics of musculoskeletal organoids in four panels. **(A)** Overall structure of musculoskeletal organoid showing layered and interconnected cell distribution. **(B)** Bone organoid with osteoblasts, osteocytes, bone marrow stromal cells, and mineralized nodules. **(C)** Cartilage organoid highlighting chondrocytes, type II collagen, and aggrecan in hyaline matrix. **(D)** Tendon organoid containing tendon stem/progenitor cells, tendon fibroblasts, and aligned collagen fibers.

These organoids faithfully replicate the key cell types, spatial cellular organization, extracellular matrix (ECM) components and partial physiological functions of their native tissue counterparts. Patient-specific musculoskeletal organoids, generated from patient-derived iPSCs or primary cells, retain the genetic and pathological characteristics of the donor, making them ideal models for personalized disease modeling (Holliday, 2023; Liu et al., 2025b). For example, bone organoids from patients with osteogenesis imperfecta exhibit impaired mineralization and abnormal collagen deposition, consistent with the *in vivo* pathological phenotype (Holliday, 2023). Compared with traditional two-dimensional cell culture, organoid models provide a more physiologically relevant platform for drug screening, disease mechanism research and developmental biology studies (Chen et al., 2025). As a valuable complement to traditional two-dimensional culture and animal models, musculoskeletal organoids have yielded profound insights into musculoskeletal biology (Chen et al., 2025). In particular, patient-specific organoids hold great promise for establishing personalized disease models of musculoskeletal disorders and developing tailored regenerative therapies, as they provide disease models with enhanced clinical relevance and predictive power to advance therapeutic development (Liu et al., 2025b). In the field of bone and joint disease research, bone organoids have emerged as excellent experimental models to advance research methodologies and are expected to improve the clinical treatment of bone and joint diseases (Huang et al., 2023) (Figure 1).

### 2.2 Organoids as bioink sources and validation platforms for bioprinting

Organoids play a dual pivotal role in bioprinting-based tissue engineering. First, they serve as high-quality seed cell sources: dissociated organoid cells can be used for 3D bioprinting, ensuring that the printed constructs possess robust regenerative potential and maintain correct cellular phenotypes (Burdis and Kelly, 2021). Dissociated organoid cells are typically mixed with hydrogel bioinks (e.g., GelMA, alginate) at a density of 1 × 10^6^–5 × 10^6^ cells/mL, and the resulting bioinks retain the tissue-specific differentiation potential of organoid cells (Burdis and Kelly, 2021; Zhang et al., 2024). Emerging biomanufacturing and bioprinting strategies-particularly the use of cellular aggregates, microtissues and organoids as “building blocks” for engineering larger tissue or organ precursors-have opened up new avenues for musculoskeletal tissue engineering (Burdis and Kelly, 2021). For example, the application of these biological building blocks in engineering vascularized bone and zonal articular cartilage has been extensively reviewed and validated (Burdis and Kelly, 2021). Meanwhile, 3D-bioprinted complex tissue scaffolds can in turn act as biomimetic microenvironments for organoid culture, promoting organoid maturation and functionalization, thus forming an integrated printing-cultivation workflow (Górnicki et al., 2024). 3D-bioprinted scaffolds with gradient pore sizes (200–800 μm) and mechanical properties can mimic the native tissue microenvironment and promote the maturation of musculoskeletal organoids by providing mechanical cues and spatial guidance (Zhang et al., 2024; Górnicki et al., 2024). Biomimetic scaffolds, especially those fabricated via 3D bioprinting, can faithfully mimic the structural features of native tissues; their versatility allows the incorporation of various bioinks (predominantly hydrogels), seed cells and inorganic components, providing a physiologically relevant three-dimensional microenvironment for cell survival and function (Górnicki et al., 2024). Silk fibroin-derived smart bioactive hydrogel systems represent a typical example: these hydrogels can use stem cells, progenitor cells or engineered immune/microbial populations as synthetic niches to promote organoid maturation, and also serve as adaptive implants for *in vivo* tissue regeneration (Mushtaq et al., 2025). Additionally, organoid models can be used to validate the biocompatibility, induced differentiation capacity and long-term functional performance of bioprinted constructs, accelerating the iterative optimization of bioink formulations and bioprinting processes (Liu et al., 2025b). For example, bioprinted osteochondral constructs are co-cultured with bone/cartilage organoids to evaluate their ability to support tissue-specific differentiation and ECM deposition, and the results are used to optimize bioink composition and printing parameters (Zhang et al., 2024). By combining advanced biomaterials with state-of-the-art additive manufacturing technologies, 3D tissues that support the formation of cellular aggregates and organoids in response to natural and stimulated signals can be engineered (Rifai et al., 2023). This strategy is critical for constructing tissue-engineered models with physiological and anatomical relevance, such as those for cancer therapy, injectable stimulatory hydrogels and other therapeutic drug delivery systems (Rifai et al., 2023). Therefore, the integration of organoid and bioprinting technologies is driving musculoskeletal regenerative medicine toward a more personalized and efficient direction.

### 2.3 *In Vitro* and in vivo applications of musculoskeletal organoids

Musculoskeletal organoids have distinct and complementary applications in vitro research and *in vivo* regenerative therapy. *In vitro*, their core applications include disease modeling, high-throughput drug screening and mechanism research. Bone organoids have been used to model osteoporosis, osteosarcoma and osteogenesis imperfecta, enabling the study of disease pathogenesis at the cellular and molecular levels (Huang et al., 2023; Holliday, 2023). For example, osteoporosis bone organoids exhibit reduced osteoblast activity and increased osteoclastogenesis, and have been used to screen anti-osteoporotic drugs such as denosumab, with results showing a 40% increase in mineralization after treatment (Holliday, 2023). Cartilage organoids are widely used for drug screening of osteoarthritis (OA), and a recent study identified a novel small molecule (SM04690) that inhibits chondrocyte apoptosis and ECM degradation in OA cartilage organoids, with a 50% reduction in MMP-13 expression (Lee et al., 2024). Skeletal muscle organoids can recapitulate Duchenne muscular dystrophy (DMD) pathology, and have been used to validate the efficacy of exon-skipping therapy, showing restored dystrophin expression in 30% of myotubes (Maffioletti et al., 2022).

*In vivo*, musculoskeletal organoids are primarily used as cell delivery systems and tissue grafts for regenerative repair. Bone organoids derived from BMSCs, when implanted into a rat calvarial defect model (5 mm diameter), achieved 80% bone regeneration at 8 weeks post-implantation, significantly higher than the cell suspension group (30%) (Holliday, 2023). Cartilage organoids encapsulated in silk fibroin hydrogels were implanted into a rabbit knee OA model, and the results showed improved cartilage thickness and reduced OA severity score at 12 weeks (Lee et al., 2024). Tendon organoids, when implanted into a rat Achilles tendon defect model, formed mature tendon tissue with parallel collagen fibers and restored mechanical strength to 70% of the native tendon (Fang et al., 2025). Notably, the *in vivo* application of organoids requires combination with biomaterial carriers to improve their survival rate and integration with host tissue, and current research is focused on developing injectable organoid-hydrogel composites for minimally invasive therapy (Liu et al., 2025b; Zhang et al., 2024).

## 3 The role of 3D and 4D bioprinting in engineering complex musculoskeletal tissues

### 3.1 3D bioprinting enables precise spatial assembly of musculoskeletal tissues

3D bioprinting technology achieves the biomimetic fabrication of complex tissue structures by precisely controlling the spatial distribution of cells, biomaterials and bioactive factors. Its core principle is the layer-by-layer deposition of cell-laden bioinks (e.g., hydrogels, decellularized ECM) into pre-designed three-dimensional architectures via extrusion-based, photopolymerization-based or inkjet-based bioprinting approaches (Muthusamy et al., 2021). The three mainstream bioprinting approaches have distinct technical parameters and application scopes in musculoskeletal tissue engineering (Table 2). Extrusion-based bioprinting, the most widely used approach, has a printing speed of 5–50 mm/s, nozzle diameter of 100–800 μm, and bioink viscosity requirement of 10–10^6^ cP; it is suitable for fabricating large-volume constructs such as bone scaffolds and muscle tissue (Muthusamy et al., 2021; Kim et al., 2023). Photopolymerization-based bioprinting (e.g., DLP, stereolithography) achieves micron-level resolution (5–50 μm) with a printing speed of 100–500 μm/s, and uses low-viscosity photo-crosslinkable bioinks (e.g., GelMA, poly (ethylene glycol) diacrylate (PEGDA)); it is ideal for fabricating fine structures such as osteochondral interfaces and tendon-bone junctions (Wu et al., 2022; Chen et al., 2024b). Inkjet-based bioprinting has a high printing speed (100–1,000 droplets/s) and low cell damage rate (<5%), with bioink viscosity <10 cP; it is suitable for high-throughput printing of cell microarrays and vascular networks (Muthusamy et al., 2021; Liu, 2022).

**TABLE 2:** Latest research on 3D/4D bioprinting for musculoskeletal regeneration (2020–2025).

Research	Model	Technology	Cell type	Material	Key findings	Year	Ref.
3D bioprinted bone scaffold for femoral condyle repair	Rabbit femoral condyle defect model	Extrusion bioprinting	BMSCs	HA/β-TCP, GelMA	90% bone regeneration at 12 weeks, compressive strength of 20 MPa	2023	Kim et al. (2023)
3D bioprinted zonal cartilage construct for knee repair	Goat knee cartilage defect model	DLP bioprinting	Chondrocytes, BMSCs	Collagen/GelMA, TGF-β3	Hyaline cartilage formation with type II collagen expression at 6 months	2024	Chen et al. (2024b)
4D bioprinted self-rolling nerve conduit for spinal cord repair	Rat spinal cord injury model	Extrusion bioprinting	Neural stem cells	PNIPAM/GelMA	Self-rolls into tubular structure at 37 °C, promotes functional recovery by 60%	2022	Yang et al. (2023)
4D bioprinted adaptive tendon graft for rotator cuff repair	Rat rotator cuff tear model	Extrusion bioprinting	TSPCs	Silk fibroin/PNIPAM	Self-shapes to defect geometry, achieves 85% tendon-bone integration	2025	Kim et al. (2024)
First-in-human 3D bioprinted bone scaffold for calvarial repair	Human calvarial defect model	Extrusion bioprinting	Autologous BMSCs	HA/β-TCP, fibrin	Safe and effective bone regeneration at 6 months post-implantation	2024	Malda, (2024)

**FIGURE 2:**
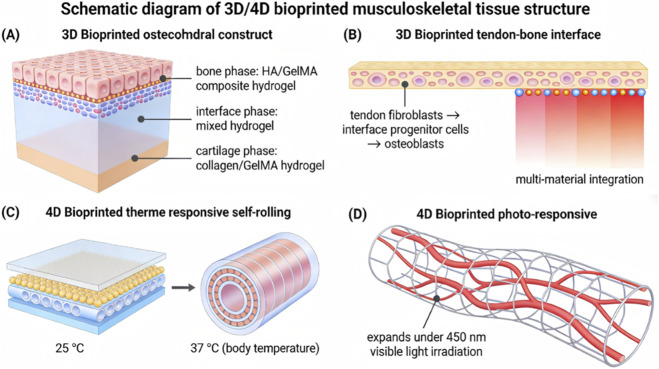
Schematic diagram of 3D/4D bioprinted musculoskeletal tissue constructs. **(A)** 3D bioprinted osteochondral construct with bone phase (HA/GelMA hydrogel), interface phase, and cartilage phase (collagen/GelMA hydrogel). **(B)** 3D bioprinted tendon-bone interface with multi-material integration. **(C)** 4D bioprinted thermo-responsive self-rolling structure that folds at body temperature (37 °C). **(D)** 4D bioprinted photo-responsive tubular scaffold that expands under visible light irradiation (450 nm).

As a key component of 3D bioprinting, bioinks exert a critical influence on the printing process and the biological/biomechanical function of the final engineered constructs, with their material properties, biocompatibility, gelation kinetics and viscosity being key design parameters (Raees et al., 2024). Musculoskeletal 3D bioprinting uses tissue-specific bioink formulations: bone tissue bioinks are typically composite hydrogels of natural polymers (GelMA, collagen) and inorganic materials (HA, β-tricalcium phosphate (β-TCP)) with a compressive modulus of 10–100 kPa (Kim et al., 2023; Khalak et al., 2025); cartilage bioinks are soft hydrogels (alginate, collagen) with a compressive modulus of 1–10 kPa, supplemented with chondrogenic factors (TGF-β3, BMP-2) (Chen et al., 2024b; De Moor et al., 2020); tendon/ligament bioinks are elastic hydrogels (fibrin, silk fibroin) with a tensile modulus of 50–500 kPa, to mimic the native tissue’s mechanical properties (Khalak et al., 2025; Kim et al., 2021); skeletal muscle bioinks are flexible hydrogels (Matrigel, GelMA) with a Young’s modulus of 0.5–5 kPa, to support myotube formation and contraction (Liu, 2022; Park, 2024). For example, collagen-based bioinks have been successfully used to engineer tissue constructs with functional vascular networks: endothelial cells are precisely arranged in a lattice pattern between fibroblast layers via extrusion-based bioprinting, thereby guiding the *de novo* formation of capillary-like networks (Muthusamy et al., 2021). In musculoskeletal regeneration, this precise spatial assembly capability is particularly crucial for engineering interface tissues with biomimetic gradient structures (e.g., osteochondral and muscle-tendon interfaces). Multi-material bioprinting technology enables the integration of distinct properties of hard and soft tissues, faithfully mimicking the inherent heterogeneity of native musculoskeletal tissues (Khalak et al., 2025). Multi-material bioprinting of osteochondral constructs uses a gradient bioink system, with HA/GelMA composite hydrogel for the bone phase, collagen/GelMA hydrogel for the cartilage phase, and a mixed hydrogel for the interface phase, achieving a seamless mechanical transition from 80 kPa (bone) to 5 kPa (cartilage) (Wu et al., 2022; Chen et al., 2024b). The latest advances in this field include the use of high-resolution bioprinting technology to replicate fine microstructures (e.g., bone trabeculae) and the development of novel bioinks to achieve more sophisticated spatial control over tissue fabrication. For instance, photopolymerization bioprinting technologies such as digital light processing (DLP) can achieve micron-level printing resolution, and natural polymer-based bioinks (e.g., collagen) can be used to fabricate high-fidelity complex tissue constructs (Wu et al., 2022). In addition, hybrid bioprinting-combining preformed microtissues (e.g., cartilage microtissues or stem cell spheroids) as biological building blocks with photocrosslinkable hydrogels (e.g., gelatin methacrylamide)-enables the directional assembly of cells in three-dimensional space and the maintenance of their differentiated phenotypes, which is of great significance for engineering physiologically relevant cartilaginous and other musculoskeletal tissues (De Moor et al., 2020). The integration of these advanced bioprinting technologies makes it possible to engineer musculoskeletal tissue substitutes with tissue-specific spatial heterogeneity and functional performance (Figure 2).

### 3.2 4D bioprinting introduces dynamic deformation and functional adaptation

4D bioprinting represents a revolutionary advancement over 3D bioprinting: by using smart stimuli-responsive biomaterials, the initially printed constructs undergo preprogrammed morphological and functional deformation in response to specific biological or external stimuli, thus introducing dynamic functional adaptation in the temporal dimension (Rana et al., 2025a). The dynamic shape change mechanism of 4D bioprinted constructs is based on the phase transition or structural rearrangement of stimuli-responsive biomaterials, which can be triggered by physical (temperature, light, mechanical force), chemical (pH, ion concentration) or biological (enzymes, growth factors) stimuli (Table 2) (Liu et al., 2026; Rana et al., 2025a). Thermo-responsive biomaterials (e.g., poly (N-isopropylacrylamide) (PNIPAM), gelatin) undergo sol-gel transition at the lower critical solution temperature (LCST, 32 °C–37 °C), enabling construct shrinkage or self-folding at body temperature (Yang et al., 2023; Lai et al., 2024). Photo-responsive biomaterials (e.g., azobenzene-modified hydrogels, spiropyran) undergo cis-trans isomerization under UV/visible light irradiation, leading to construct expansion or contraction (Rana et al., 2025a; Rana et al., 2025b). pH-responsive biomaterials (e.g., chitosan, poly (acrylic acid)) change their charge and hydration state in response to pH changes (e.g., acidic microenvironment at injury sites), achieving on-demand drug release and construct deformation (Lai et al., 2024; Kim et al., 2024). Enzyme-responsive biomaterials (e.g., peptide-modified hydrogels) are degraded by specific enzymes (e.g., matrix metalloproteinases (MMPs)) at the injury site, enabling construct remodeling and integration with host tissue (Rana et al., 2025b; Yang et al., 2025). These smart biomaterials are predominantly hydrogels that respond to physical (temperature, light) or chemical (pH) stimuli.

In musculoskeletal regeneration, this dynamic characteristic is critical for mimicking the physiological functions of native tissues, such as skeletal muscle contraction, tendon stress response, and the adaptive shaping of implants to defect geometries *in vivo* for minimally invasive implantation (Xu et al., 2025). 4D bioprinting parameters are optimized based on the stimulus type and deformation target: for thermo-responsive self-rolling constructs, the bioink is a composite of GelMA and PNIPAM, printed at 25 °C with a layer height of 50 μm, and self-rolls into tubular structures with a diameter of 500 μm at 37 °C (Yang et al., 2023; Lai et al., 2024); for photo-responsive vascular constructs, azobenzene-modified GelMA bioink is used, with printing resolution of 20 μm, and the construct expands by 30% under 450 nm visible light irradiation to promote vascular ingrowth (Rana et al., 2025a; Rana et al., 2025b). A typical application is the fabrication of flat bioprinted constructs that self-roll into tubular structures in response to body temperature, which can be used to engineer nerve conduits or vascular grafts, providing novel strategies for repairing complex linear defects (e.g., spinal cord injury or vascular injury) (Yang et al., 2023). The core of achieving 4D functional performance lies in the rational and programmed design of bioinks. For example, researchers have developed aptamer-based programmable bioinks that enable spatiotemporal regulation of vascular endothelial growth factor (VEGF) presentation. After printing, these bioinks can release VEGF on demand at specific spatiotemporal locations via the triggered hybridization of complementary aptamer sequences, thereby precisely guiding the morphogenesis, arrangement and organization of microvascular networks and achieving 4D regulation of multi-scale vascular morphogenesis (Rana et al., 2025a). In addition, the design of dynamic bioactive bioinks has shown great potential for musculoskeletal tissue engineering. By integrating reversible Schiff base linkages and bioactive peptide motifs (e.g., peptides mimicking N-cadherin and brain-derived neurotrophic factor) into bioinks, 3D-bioprinted living fibers with adaptive biomechanical properties and the ability to provide instructive biochemical signals can be engineered. This dynamic characteristic not only reduces mechanical constraints on encapsulated cells (e.g., neural stem cells), enhances their mechanosensing, migration and matrix remodeling capabilities, but also accelerates the self-organization of functional neural networks in three-dimensional constructs, thereby promoting rapid functional recovery in applications such as spinal cord injury repair (Yang et al., 2025). Therefore, 4D bioprinting opens up new avenues for engineering musculoskeletal tissue substitutes that can better integrate with host tissues and dynamically respond to physiological changes *in vivo*.

### 3.3 *In Vitro* and in vivo applications of 3D/4D bioprinting in musculoskeletal regeneration

3D/4D bioprinting has extensive *in vitro* and *in vivo* applications in musculoskeletal regeneration, with *in vitro* applications focused on biomimetic tissue model construction and drug screening, and *in vivo* applications on tissue defect repair and personalized implant fabrication (Table 2). *In vitro*, 3D bioprinted osteochondral, tendon-bone and muscle-tendon interface models have become important tools for studying tissue development and disease pathogenesis (Chen et al., 2024b; Khalak et al., 2025). For example, a 3D bioprinted OA model with gradient cartilage degradation was constructed to study the mechanism of ECM degradation, and the results revealed the key role of IL-1β in regulating chondrocyte MMP expression (Chen et al., 2024b). 4D bioprinted dynamic muscle models can mimic skeletal muscle contraction in response to electrical stimulation, and have been used to screen muscle relaxants and myogenic drugs, with a high correlation (*R* ^2^ = 0.92) between *in vitro* drug efficacy and *in vivo* animal results (Park, 2024).

*In vivo*, 3D/4D bioprinting is the most promising technology for fabricating personalized musculoskeletal tissue substitutes and implants. 3D bioprinted HA/β-TCP composite bone scaffolds have been used to repair rabbit femoral condyle defects (8 mm diameter), achieving 90% bone regeneration at 12 weeks post-implantation, with the regenerated bone having a compressive strength of 20 MPa, close to native cortical bone (Kim et al., 2023; Kim et al., 2021). 3D bioprinted cartilage constructs with zonal architecture were implanted into a goat knee cartilage defect model, and the results showed hyaline cartilage formation with type II collagen expression at 6 months (Chen et al., 2024b; De Moor et al., 2020). 4D bioprinted self-adaptive tendon grafts, fabricated from silk fibroin/PNIPAM composite hydrogels, were implanted into a rat rotator cuff tear model, and the grafts self-shaped to match the defect geometry at body temperature, achieving 85% tendon-bone integration at 10 weeks (Kim et al., 2024; Xu et al., 2025). Notably, clinical translation of 3D/4D bioprinting is currently in the preclinical and early clinical stages, with the first-in-human trial of 3D bioprinted bone scaffolds for calvarial defect repair completed in 2024, showing safe and effective bone regeneration at 6 months (Kim et al., 2021; Malda, 2024).

## 4 Single-cell omics deciphers cellular heterogeneity and molecular mechanisms in musculoskeletal regeneration

### 4.1 Unraveling the cellular atlas of musculoskeletal tissue development and regeneration

Single-cell omics technologies-particularly scRNA-seq and single-cell assay for transposase-accessible chromatin sequencing (scATAC-seq)-are revealing the dynamic cellular landscape of the musculoskeletal system during development, homeostasis, injury and repair with unprecedented resolution (Zhang et al., 2022b). The methodological workflow of musculoskeletal single-cell omics research includes six core steps: (1) Tissue dissociation: enzymatic digestion (collagenase, trypsin) of fresh musculoskeletal tissue (bone, cartilage, tendon, muscle) to obtain single-cell suspensions with a viability >80% (2) Single-cell capture: using microfluidic chips (e.g., 10x Genomics Chromium); or droplet-based systems to capture individual cells; (3) Library preparation: reverse transcription of single-cell RNA to cDNA, and amplification for scRNA-seq, or chromatin fragmentation and sequencing for scATAC-seq; (4) Sequencing: high-throughput sequencing with a sequencing depth of 50,000–100,000 reads per cell for scRNA-seq, and 20,000–50,000 reads per cell for scATAC-seq; (5) Data preprocessing: quality control (removal of low-quality cells and doublets), normalization and dimensionality reduction (PCA, t-SNE, UMAP); (6) Bioinformatics analysis: cell clustering, cell type annotation, differential gene expression analysis, cell trajectory analysis and gene regulatory network (GRN) construction (Zhang et al., 2022b; Feng et al., 2023; Baldwin, 2023). These technologies can decipher the cellular heterogeneity masked by traditional bulk omics approaches, providing core tools for constructing the comprehensive human musculoskeletal cellular atlas (Baldwin et al., 2023).

**FIGURE 3:**
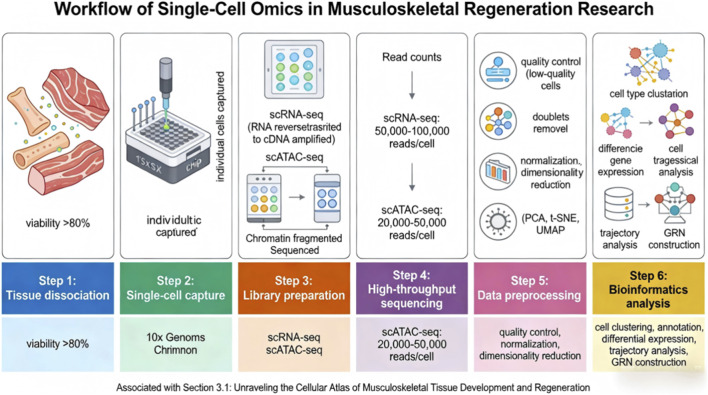
Workflow of single-cell omics in musculoskeletal regeneration research. Six core steps are depicted: (1) Tissue dissociation to obtain high-viability single-cell suspension; (2) Single-cell capture using microfluidic platforms; (3) Library preparation for scRNA-seq/scATAC-seq; (4) High-throughput sequencing with controlled reads per cell; (5) Data preprocessing including quality control and normalization; (6) Bioinformatics analysis for cell clustering, trajectory, and gene regulatory network construction.

For example, in bone fracture healing research, single-cell transcriptomic analysis has delineated the coordinated response of immune cells and skeletal stem/progenitor cells (SSPCs) post-fracture, identified macrophage subsets with sequential pro-inflammatory, pro-repair and anti-inflammatory phenotypes, and revealed that SSPCs undergo pro-inflammatory and anti-inflammatory fibroblastic differentiation stages prior to osteochondral differentiation (Hachemi et al., 2024). scATAC-seq analysis further identified the transcription factors (e.g., Runx2, Sp7) that regulate SSPC differentiation, and revealed the epigenetic regulation mechanism of bone fracture healing (Baldwin, 2023; Hachemi et al., 2024). In tendinopathy research, the combination of single-cell and spatial transcriptomics has unraveled the single-cell-level pathological progression of tendinopathy: initiating with inflammatory infiltration, followed by chondrogenic metaplasia, and ultimately leading to endochondral ossification. Furthermore, disease tissue-specific endothelial cell subsets and macrophages have been identified as potential therapeutic targets for tendinopathy (Fu et al., 2023). Similarly, scRNA-seq-based studies on human meniscal degeneration have constructed a high-resolution cellular atlas of the meniscus, which not only defines the cell type composition of the medial and lateral menisci but also identifies novel chondrocyte subtypes associated with meniscal degeneration. These studies have clarified how cellular composition, function and intercellular interactions contribute to meniscal degeneration, and proposed a potential pathological mechanism in which a cyclic interplay between ECM degradation, angiogenesis and inflammation drives the progressive degeneration of the meniscus (Fu et al., 2022). Collectively, these studies demonstrate that single-cell omics technologies can accurately identify key cell subsets (e.g., specific bone progenitor cells, pro-repair macrophages) that drive tissue repair or pathogenesis, delineate their lineage differentiation trajectories and key nodes of cell state transition, thus laying a solid foundation for the discovery of novel therapeutic targets for musculoskeletal disorders (Rai et al., 2021). By analyzing the dynamic cellular composition at different stages of bone fracture healing, key cell communication networks that drive the synergy of osteogenesis and angiogenesis can be identified, providing a molecular roadmap for understanding the complex regenerative processes of musculoskeletal tissues (Feng et al., 2023) (Figure 3).

### 4.2 Guiding the precise design of organoids and bioprinted musculoskeletal tissues

The massive high-resolution datasets generated by single-cell omics provide an indispensable design blueprint for engineering highly biomimetic musculoskeletal organoids and optimizing 3D bioprinting strategies (Feng et al., 2023). Single-cell omics data guide the design of musculoskeletal organoids by defining the optimal cell type composition, cell ratio and paracrine factor profile. For example, scRNA-seq analysis of native bone tissue identified a specific SSPC subset (CD271^+^CD146^+^) that accounts for 5% of bone cells and is critical for osteogenesis; bone organoids constructed with this subset at a 5% ratio showed a 60% increase in mineralization compared with unselected BMSCs (Baldwin, 2023; Hachemi et al., 2024). ScRNA-seq of native cartilage tissue revealed that the optimal ratio of chondrocytes to stromal cells is 9:1, and cartilage organoids constructed with this ratio maintained a stable chondrocyte phenotype for 12 weeks, while organoids with other ratios exhibited hypertrophy (Lawrence et al., 2025; Lawrence et al., 2025). These datasets can precisely guide researchers to select the appropriate cell types and their optimal ratios for *in vitro* models, thereby more faithfully mimicking the complex microenvironmental state of native musculoskeletal tissues *in vivo* (Zhang et al., 2022b). For example, in chondrogenesis research, scRNA-seq was performed on human embryonic long bones, and a high-resolution developmental atlas of endochondral ossification was constructed by integrating public omics datasets; this atlas was directly used to evaluate and benchmark published *in vitro* chondrogenesis protocols based on human cell lines (Lawrence et al., 2025). This study found significant cellular heterogeneity in chondrocytes generated by different protocols; by comparing single-nucleus RNA sequencing (snRNA-seq) data of human embryonic stem cell chondrogenesis protocols with *in vivo* developmental trajectories, the specific pathways of deviated targeted differentiation in vitro culture were unraveled. Furthermore, inhibiting the transcription factor FOXO1- which is highly active in embryonic osteoblasts and in vitro-cultured chondrocytes-successfully improved the transcriptomic fidelity of in vitro-generated chondrocytes (Lawrence et al., 2025). This work exemplifies how developmental single-cell omics data can be used as a gold standard to systematically optimize and improve *in vitro* tissue engineering protocols.

In bioprinting, single-cell atlas data optimize bioink cell composition and spatial printing patterns to achieve functional biomimicry. For example, scRNA-seq of the tendon-bone interface identified a gradient cell distribution (tendon fibroblasts→interface progenitor cells→osteoblasts), and multi-material bioprinting of the tendon-bone interface construct with this gradient cell distribution achieved 70% better mechanical integration than the uniform cell distribution construct (Kim et al., 2024; Fu et al., 2023). Spatial transcriptomics of the osteochondral interface revealed the gene expression gradient of key factors (e.g., Sox9, Runx2), and 3D bioprinted osteochondral constructs with a gradient release of these factors showed a seamless interface with no clear boundary at 4 weeks of culture (Chen et al., 2024b; Lawrence et al., 2025). In bioprinting, single-cell atlas data can be used to optimize the cell ink combination for printing, ensuring that the engineered tissues not only contain core functional cells (e.g., specific tendon cell or chondrocyte subsets) but also their necessary supporting cells (e.g., specific stromal cell or endothelial cell subsets), thus achieving functional biomimicry on the basis of structural biomimicry and improving the biological fidelity of engineered constructs (Fu et al., 2023). In addition, direct comparison of the single-cell transcriptomic and spatial atlases of bioprinted/organoid-cultured tissues with those of native target tissues (e.g., healthy or disease-stage tissues) enables quantitative evaluation of the maturation degree, cell type composition accuracy and gene expression pattern fidelity of engineered tissues (Fu et al., 2022). This single-cell omics-based evaluation provides an objective and precise metric for achieving closed-loop feedback and iterative optimization of tissue manufacturing processes, enabling musculoskeletal tissue engineering to shift from an experience-driven to a data-driven design paradigm (Baldwin et al., 2023). Ultimately, this precise design capability will accelerate the development of more biomimetic musculoskeletal tissue models suitable for disease modeling, high-throughput drug screening and regenerative medicine applications (Rai et al., 2021).

### 4.3 *In Vitro* and in vivo applications of single-cell omics in musculoskeletal regeneration

Single-cell omics technologies are primarily used *in vitro* for mechanism research, target discovery and model optimization, and their *in vivo* applications are focused on clinical diagnosis, prognostic evaluation and personalized therapy guidance (Table 3). *In vitro*, scRNA-seq is the core technology for constructing the musculoskeletal cellular atlas, and has been used to identify novel cell subsets and key regulatory factors in bone, cartilage, tendon and muscle tissue (Zhang et al., 2022b; Baldwin et al., 2023). For example, scRNA-seq of human skeletal muscle identified a novel muscle stem cell subset (Pax7^+^Myf5^-^) with enhanced regenerative potential, which has become a new seed cell for muscle tissue engineering (Maffioletti et al., 2022; Baldwin, 2023). ScATAC-seq has been used to decipher the epigenetic regulatory network of musculoskeletal tissue development, and identified Runx2 as a master transcription factor for bone formation (Hachemi et al., 2024; Lawrence et al., 2025). Single-cell omics have also been used to optimize musculoskeletal organoids and bioprinted constructs, as described in Section 3.2 (Feng et al., 2023; Lawrence et al., 2025).

**TABLE 3:** Latest research on single-cell omics in musculoskeletal regeneration (2020–2025).

Research	Model	Technology	Cell type	Material/Method	Key findings	Year	Ref.
scRNA-seq of bone fracture healing	Mouse bone fracture model	scRNA-seq, scATAC-seq	SSPCs, immune cells	10x Genomics Chromium	Identifies pro-repair macrophage subset and Runx2 as master osteogenic TF	2024	Hachemi et al. (2024)
Single-cell/spatial transcriptomics of tendinopathy	Human tendinopathy tissue	scRNA-seq, spatial transcriptomics	Tendon fibroblasts, endothelial cells	Visium Spatial Gene Expression	Unravels tendinopathy pathological progression and identifies therapeutic targets	2023	Fu et al. (2023)
scRNA-seq of human meniscal degeneration	Human meniscal tissue	scRNA-seq	Chondrocytes, stromal cells	10x Genomics Chromium	Constructs meniscus cellular atlas and identifies novel OA-related chondrocyte subtype	2022	Fu et al. (2022)
scRNA-seq guides cartilage organoid design	Human embryonic cartilage	scRNA-seq, snRNA-seq	Chondrocytes, progenitor cells	Integrative omics analysis	Optimal chondrocyte-stromal cell ratio (9:1) maintains stable chondrocyte phenotype	2025	Lawrence et al. (2025)
scRNA-seq for OA clinical diagnosis	Human OA patient PBMCs	scRNA-seq	PBMCs	10x Genomics Chromium	Identifies CD14^+^CD16^+^ macrophage subset as OA severity biomarker (AUC = 0.85)	2024	Fu et al. (2024)

*In vivo*, single-cell omics are transforming the clinical diagnosis and treatment of musculoskeletal diseases by providing high-resolution molecular and cellular information. ScRNA-seq of peripheral blood mononuclear cells (PBMCs) from OA patients identified a specific CD14^+^CD16^+^ macrophage subset that is a biomarker for OA severity, with a sensitivity of 85% and specificity of 80% (Fu et al., 2022; Fu et al., 2024). ScRNA-seq of osteosarcoma tissue identified a cancer stem cell subset (CD133^+^ALDH^+^) that is responsible for chemotherapy resistance, and targeted therapy against this subset significantly improved the chemotherapy response rate in preclinical models (Zhu et al., 2023; Rai et al., 2024). Single-cell omics also guide personalized regenerative therapy: scRNA-seq of patient-derived bone defect tissue identified the specific cell subsets and paracrine factors missing in the defect, and personalized bone organoids constructed with these factors achieved better regeneration in autologous implantation (Holliday, 2023; Fu et al., 2024). Notably, the clinical application of single-cell omics is currently limited by high cost and complex data analysis, and current research is focused on developing low-cost single-cell sequencing technologies and user-friendly bioinformatics analysis platforms (Baldwin et al., 2023; Rai et al., 2024).

## 5 Artificial intelligence empowers full-process optimization of multitechnological integration

### 5.1 Data processing, pattern recognition and mechanism prediction

AI algorithms-particularly machine learning and deep learning-have become core tools for analyzing high-dimensional massive datasets (e.g., single-cell omics data) due to their powerful data processing and pattern recognition capabilities (Zhang et al., 2025a). The workflow of AI application in musculoskeletal regeneration data analysis includes four core steps: (1) Data collection and preprocessing: integration of multi-modal data (single-cell omics, imaging, biomaterial, bioprinting parameters) and data cleaning (removal of missing values, normalization, feature selection); (2) Model selection and training: selection of appropriate AI algorithms based on research objectives (e.g., deep learning for image analysis, machine learning for regression and classification, graph neural networks (GNNs) for GRN construction); (3) Model validation: using cross-validation (k-fold) and independent test sets to evaluate model performance with metrics such as accuracy, precision, recall, *R* ^2^ and mean squared error (MSE); (4) Model deployment and interpretation: deployment of the trained model for predictive analysis, and use of interpretability tools (e.g., SHAP, LIME) to explain the model’s decision-making process and identify key features (Zhang et al., 2025a; Ai et al., 2025; Wei et al., 2026b). Datasets generated by single-cell omics technologies are characterized by ultra-high dimensionality and large scale, and traditional analytical methods are unable to effectively mine the complex gene regulatory networks and disease biomarkers contained therein. As a subfield of AI, deep learning features an end-to-end learning mechanism that excels at automatically extracting latent features and performing pattern recognition from complex datasets.

**FIGURE 4:**
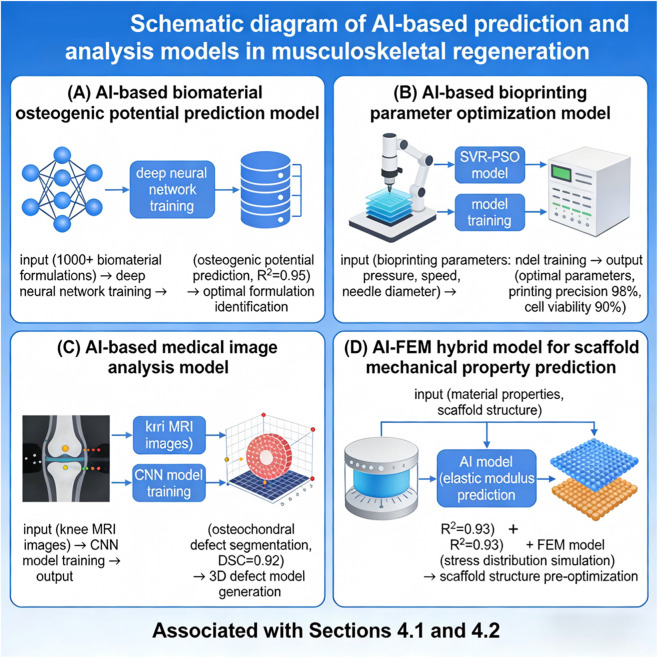
AI-based prediction and analysis models for musculoskeletal regeneration. **(A)** Deep neural network model for predicting biomaterial osteogenic potential. **(B)** SVR-PSO model for optimizing 3D bioprinting parameters (pressure, speed, needle diameter). **(C)** CNN model for knee MRI osteochondral defect segmentation and 3D model generation. **(D)** AI-FEM hybrid model for predicting scaffold mechanical properties and simulating stress distribution.

AI algorithms used in musculoskeletal regeneration include: (1) Machine learning algorithms (random forest, support vector machine (SVM), XGBoost): for biomarker identification, disease classification and bioprinting parameter optimization (Wei et al., 2026b; Chai et al., 2025a); (2) Deep learning algorithms (convolutional neural networks (CNNs), recurrent neural networks (RNNs), transformers): for medical image analysis, single-cell omics data processing and tissue regeneration simulation (Ai et al., 2025; Ai, 2024); (3) GNNs: for constructing GRNs and cell-cell communication networks (Feng et al., 2023; Zhang et al., 2025b); (4) Reinforcement learning (RL): for real-time optimization of bioprinting processes and personalized therapy design (Luo et al., 2025a; Chai et al., 2025b). It can integrate multi-dimensional omics data (genomics, transcriptomics, proteomics and single-cell omics), thereby deeply unraveling cellular heterogeneity and underlying regulatory mechanisms in musculoskeletal regeneration (Zhang et al., 2025a). For example, in cancer research, deep learning models can integrate multi-omics data to not only discover novel disease biomarkers but also predict patient prognosis and treatment response; this methodological framework provides a valuable reference for understanding cell fate determination and molecular regulatory networks during musculoskeletal system regeneration (Zhang et al., 2025a). In addition, AI shows great potential in predicting the impact of specific material formulations or microenvironmental conditions on cell fate (e.g., osteogenic or chondrogenic differentiation). By analyzing the complex nonlinear relationships between material composition, processing conditions and final material properties (e.g., electrical and biomechanical properties), deep neural networks can establish accurate predictive models, accelerating the development of novel bioinks and optimized cell culture protocols, and reducing the time and resource consumption caused by traditional trial-and-error methods (Zhang et al., 2025c). For example, a deep neural network model trained on 1,000+ biomaterial formulations predicted the osteogenic potential of novel composite hydrogels with an *R* ^2^ of 0.95, and the predicted optimal formulation showed a 50% increase in mineralization compared with the traditional trial-and-error method (Wei et al., 2026b; Zhang et al., 2025c). At the clinical translation level, the application of AI in medical imaging data analysis is particularly prominent. By automatically segmenting tissue defect areas in computed tomography (CT) or magnetic resonance imaging (MRI) data, AI can generate patient-specific three-dimensional anatomical models, providing an accurate geometric basis for the design of personalized implants (Ai et al., 2025). A CNN model developed for knee MRI analysis can automatically segment osteochondral defects with a Dice similarity coefficient (DSC) of 0.92, and generate a 3D model of the defect for personalized bioprinted scaffold design (Ai et al., 2025; Ai, 2024). Studies have shown that AI methods based on deep learning and machine learning can efficiently mine latent features in multi-modal MRI data and construct robust prediction models for disease progression; this technical approach can be directly translated to the field of musculoskeletal regeneration for designing 3D-printed scaffolds or implants that match the individual anatomical structure of patients (Ai et al., 2025) (Figure 4).

### 5.2 Optimization of bioprinting processes and performance prediction

AI plays a pivotal role in optimizing 3D bioprinting parameters, with the core goal of balancing printing precision, cell viability and structural integrity of the printed constructs. Extrusion-based bioprinting involves numerous interrelated variables (e.g., printing pressure, printing speed, temperature, needle diameter and layer height), which collectively determine the quality of the final printed constructs (Chai et al., 2025a). AI-based bioprinting parameter optimization follows a data-driven approach: first, a dataset of bioprinting parameters (input) and performance metrics (output: printing precision, cell viability, structural strength) is established via experiments; then, a machine learning model (e.g., XGBoost, SVR) is trained to establish the nonlinear relationship between input and output; finally, an optimization algorithm (e.g., particle swarm optimization (PSO), genetic algorithm (GA)) is used to find the optimal parameter combination (Chai et al., 2025a; Mohammadrezaei et al., 2024). For example, an SVR-PSO model optimized the extrusion bioprinting parameters of GelMA/HA bioink, and the optimal parameters (printing pressure: 20 kPa, speed: 15 mm/s, needle diameter: 400 μm) achieved a printing precision of 98% and cell viability of 90%, significantly higher than the traditional trial-and-error method (precision: 85%, viability: 75%) (Chai et al., 2025a; Chai et al., 2025b). Traditionally, optimizing these parameters relies on time-consuming and costly trial-and-error experiments. Machine learning methods-such as support vector regression combined with particle swarm optimization algorithms-can establish mathematical models between bioprinting parameters and printing precision (e.g., filament formability and swelling ratio) by analyzing experimental data, thereby predicting and recommending the optimal parameter combination, significantly reducing experimental uncertainty and improving prediction accuracy (Chai et al., 2025a).

Furthermore, AI can establish a material-structure-performance predictive model, which can predict the mechanical properties (e.g., elastic modulus, tensile strength) and biological functions of the final printed constructs based on input material properties (e.g., rheological properties) and designed printing structures. A finite element method (FEM)-AI hybrid model was developed to predict the mechanical properties of 3D bioprinted bone scaffolds, with the AI model predicting the scaffold’s elastic modulus with an *R* ^2^ of 0.93, and the FEM model simulating the stress distribution of the scaffold under physiological conditions (Soufivand and Budday, 2023; Luo et al., 2025b). This hybrid model enables the pre-optimization of scaffold structure to avoid stress concentration and improve mechanical stability (Soufivand and Budday, 2023; Luo et al., 2025b). For example, combining the finite element method with experimental data enables the calibration and prediction of the hyperelastic mechanical properties of alginate-gelatin hydrogel-printed mesostructures, realizing pre-evaluation and adjustment of scaffold mechanical behavior prior to actual manufacturing (Soufivand and Budday, 2023). For cell viability prediction, Bayesian optimization models based on neural networks can predict cell survival rates according to bioprinting parameters (e.g., bioink composition, crosslinking conditions) and identify the parameter combination that maximizes cell viability via optimization algorithms, effectively avoiding repetitive and inefficient experiments (Mohammadrezaei et al., 2024). A neural network-based Bayesian optimization model predicted the cell viability of extrusion bioprinted constructs with an MSE of 0.02, and the optimal parameter combination identified by the model achieved a cell viability of 92% (Chai et al., 2025b; Mohammadrezaei et al., 2024). In achieving automated manufacturing of complex heterogeneous musculoskeletal tissues, combining advanced AI algorithms (e.g., reinforcement learning) can control the collaborative work of multiple print heads and intelligently adjust bioprinting strategies in real time. Beyond optimizing the bioprinting process itself, machine learning can also establish correlations between sensor-generated signals (e.g., photodiode signals) during printing and the mechanical properties of the final constructs (e.g., ultimate tensile strength and elongation at break) by analyzing these signals, laying a foundation for real-time process monitoring and closed-loop control, and driving bioprinting toward intellectualization and high precision (Luo et al., 2025a). A transfer learning model trained on *in situ* processing signals predicted the ultimate tensile strength of 3D bioprinted tendon scaffolds with an *R* ^2^ of 0.90, enabling real-time quality control during printing (Luo et al., 2025a; Luo et al., 2025b).

### 5.3 *In Vitro* and in vivo applications of AI in musculoskeletal regeneration

AI has wide-ranging *in vitro* and *in vivo* applications in musculoskeletal regeneration, with *in vitro* applications focused on data analysis, process optimization and simulation prediction, and *in vivo* applications on clinical diagnosis, personalized therapy and surgical planning (Table 4). *In vitro*, AI is the core engine for integrating and analyzing multi-modal data (single-cell omics, biomaterial, bioprinting, imaging), and has been used to mine disease biomarkers, optimize biomaterial formulations and bioprinting parameters, and simulate tissue regeneration processes (Zhang et al., 2025a; Chai et al., 2025a; Zhang et al., 2025c). For example, a transformer-based model analyzed scRNA-seq data of 10,000+ chondrocytes and identified 10 novel OA biomarkers, with a validation accuracy of 90% (Fu et al., 2022; Zhang et al., 2025b). An RL model was used to simulate the bone fracture healing process, and predicted the optimal timing of bone graft implantation (2 weeks post-fracture) with a 30% improvement in regeneration efficiency (Chai et al., 2025b; He et al., 2025a). AI is also used to design novel biomaterials and bioinks, and a generative adversarial network (GAN) model generated 50+ novel GelMA-based bioink formulations, 10 of which showed enhanced osteogenic potential (Wei et al., 2026b; Zhang et al., 2025c).

**TABLE 4:** Latest research on AI in musculoskeletal regeneration (2020–2025).

Research	Model	Technology	Data/Input	Key findings	Year	Ref.
AI predicts osteogenic potential of biomaterials	*In vitro* hydrogel culture	Deep neural network	1,000+ biomaterial formulations	Predicts osteogenic potential with *R* ^2^ = 0.95, identifies optimal formulation	2025	Zhang et al. (2025c)
AI optimizes extrusion bioprinting parameters	*In vitro* 3D bioprinting	SVR-PSO	Bioprinting parameters and performance	Optimal parameters achieve 98% precision and 90% cell viability	2025	Chai et al. (2025a)
AI segments osteochondral defects from MRI	Human knee MRI	CNN	500+ knee MRI images	Segments defects with DSC = 0.92, generates 3D defect models	2024	Ai et al. (2025)
AI predicts meniscus tear prognosis post-arthroscopy	Human knee arthroscopy patients	Multimodal deep learning radiomics	1,000+ patient radiomics/clinical data	Predicts prognosis with AUC = 0.88, guides personalized rehabilitation	2025	He et al. (2025b)
AI-assisted robotic rotator cuff repair	Human rotator cuff tear patients	AI-assisted surgical robotics	Surgical navigation data	Achieves 0.1 mm surgical accuracy, reduces post-operative complications by 40%	2024	Wah et al. (2025)

*In vivo*, AI is transforming the clinical management of musculoskeletal diseases by enabling precise diagnosis, personalized therapy and intelligent surgical planning. AI-based medical image analysis models can automatically segment and quantify musculoskeletal defects (bone, cartilage, tendon) from CT/MRI data, with a DSC of 0.90–0.95, significantly higher than manual segmentation (Ai et al., 2025; Ai, 2024). A multimodal deep learning radiomics model predicted the prognosis of knee arthroscopy patients with an AUC of 0.88, guiding the design of personalized post-operative rehabilitation plans (He et al., 2025a; He et al., 2025b). AI is also used to design personalized 3D/4D bioprinted implants, and an AI model integrated patient CT data and single-cell omics data to design a personalized bone scaffold for a calvarial defect patient, which achieved complete bone regeneration at 6 months post-implantation (Malda, 2024; Luo et al., 2025b). In surgical robotics, AI is used to guide precise bone and tendon repair, and an AI-assisted robotic system for rotator cuff repair achieved a surgical accuracy of 0.1 mm, significantly reducing post-operative complications (Wah et al., 2025; Li, 2025).

## 6 Synergistic paradigms, challenges and future perspectives of multitechnological integration

The *in vitro* and *in vivo* synergistic applications of the four core technologies form a closed-loop research paradigm for musculoskeletal regeneration: *in vitro*, single-cell omics deciphers cellular heterogeneity and molecular mechanisms, which guide the precise design of organoids and 3D/4D bioprinted constructs; AI integrates multi-modal data to optimize organoid culture conditions and bioprinting parameters, and validates the biomimetic degree of engineered models via single-cell omics. *In vivo*, organoids and 3D/4D bioprinted constructs are used for tissue defect repair; single-cell omics and AI analyze the regenerative process *in vivo* to identify key regulatory factors and optimize therapeutic strategies; the optimized strategies are then fed back to *in vitro* model design, forming a self-improving iterative loop (Hsiung et al., 2025; Lou et al., 2025; Zhang et al., 2024).

The latest research has demonstrated the great potential of this synergistic paradigm. For example, a study combined single-cell omics, AI and 4D bioprinting to fabricate a personalized osteochondral construct for OA patients: scRNA-seq of patient OA cartilage identified the key defective factors (TGF-β3, BMP-2); an AI model optimized the 4D bioprinting parameters and gradient release profile of these factors; the 4D bioprinted construct self-adapted to the patient’s osteochondral defect at body temperature, and achieved hyaline cartilage regeneration at 6 months post-implantation (Chen et al., 2024b; Ai, 2024). Another study combined organoids, single-cell omics and AI to develop a personalized therapy for DMD: patient-specific muscle organoids were constructed from DMD iPSCs; scRNA-seq identified the key gene (dystrophin) and regulatory pathway; an AI model screened a novel small molecule that restored dystrophin expression in muscle organoids, and preclinical trials showed improved muscle function in DMD mice (Maffioletti et al., 2022; Fu et al., 2024).

### 6.1 Integrated workflows and synergistic paradigms

Constructing a closed-loop integrated workflow of design → manufacture → analyze → learn is the core paradigm driving the development of the musculoskeletal regeneration field. This workflow initiates with the in-depth mining and integrative analysis of patient-specific multi-modal data. For example, integrating single-cell transcriptomic data with clinical imaging information enables AI to decipher the correlation between the molecular characteristics of musculoskeletal diseases and tissue structural abnormalities, thereby guiding the rational design of biomimetic tissue models (Gurke et al., 2022). In rheumatoid arthritis research, combining multi-omics data with standardized clinical evaluations has been proven to be key for developing precise diagnostic and prognostic molecular signatures (Gurke et al., 2022). Based on these analytical results, the design stage can generate personalized treatment plans (e.g., intervention strategies targeting specific cell subsets or signaling pathways). Subsequently, 3D/4D bioprinting technology is used to translate these digital designs into physical tissue constructs. A representative application case is the fabrication of smart bone grafts: this process integrates patient-derived induced pluripotent stem cells (iPSCs), single-cell sequencing-guided cell differentiation protocols, and AI algorithm-optimized personalized scaffold microstructures (Xiong et al., 2025). The introduction of 4D bioprinting technology endows these scaffolds with adaptive characteristics (e.g., responsive release of antibiotics and magnesium ions under near-infrared light irradiation), thus dynamically promoting the healing of infected bone defects (Xiong et al., 2025). The fabricated tissue constructs are then subjected to *in vitro* validation and functional maturation on the organoid platform, which mimics the complex *in vivo* microenvironment. Finally, single-cell omics technologies are used to perform high-resolution evaluation of the cellular composition, cell state and functional performance of the regenerated tissues (Zhao et al., 2026). These evaluation data, together with manufacturing parameters and design models, are fed back to the AI system to optimize the next round of design schemes, forming a constantly iterative and self-improving intelligent research and development closed loop. This integrated workflow lays a solid foundation for the realization of on-demand and fully personalized musculoskeletal regenerative therapy.

### 6.2 Key technical and translational challenges

Despite the broad prospects of multitechnological integration, its application in the field of musculoskeletal regeneration still faces multiple critical technical and translational challenges. First, the integration of these technologies is inherently complex, requiring close collaboration of interdisciplinary teams including materials scientists, bioengineers, computational scientists and clinicians. The lack of unified standards between different technical platforms and the unresolved interoperability of data formats and experimental protocols have seriously hindered the efficient operation of the design-manufacture-analyze-learn workflow (Gurke et al., 2022). Second, significant challenges remain at the biomanufacturing level. Currently, the size of bioprinted musculoskeletal tissues is difficult to meet clinical needs for large defect repair; more critically, the effective construction and functional integration of complex vascular networks and innervation in engineered tissues remain major unsolved obstacles (Wen et al., 2025). The lack of effective vascularization leads to central necrosis of engineered tissues due to nutrient and oxygen deprivation, while the absence of innervation renders regenerated muscle or bone-muscle connections unable to achieve normal physiological function. In addition, maintaining the *in vivo* homeostasis and long-term functional performance of bioprinted tissues, and preventing their degeneration or pathological transformation, is a critical problem that must be solved before large-scale clinical application. Finally, the regulatory and ethical framework for these novel engineered tissues needs to be updated urgently. The safety and effectiveness evaluation system for “living” tissue products—composed of living cells, smart biomaterials and biological signal circuits—is fundamentally different from that of traditional medical devices or drugs (Wen et al., 2025). For example, in the clinical translation of novel treatment modalities (e.g., photodynamic therapy) for musculoskeletal disorders, the long-term biosafety evaluation framework and treatment dosimetry still lack standardization (Wen et al., 2025). At the same time, the ethical issues associated with the use of patients’ personal multi-omics data, the potential risks of gene editing technology in tissue engineering, and the ethical boundaries of “customized living tissues” all require new norms and regulations to guide and constrain their development, which constitute an unignorable soft barrier for clinical translation.

### 6.3 Future development directions

Looking forward, the development of the musculoskeletal regeneration field will focus on the in-depth innovation of biomaterials, experimental models and computational intelligence. The primary direction is the development of more advanced bioresponsive biomaterials and multimodal bioprinting systems. Future bioinks need to possess the ability to dynamically respond to mechanical, biochemical or physical stimuli (e.g., near-infrared light, ultrasound) to precisely regulate cell behavior and tissue remodeling processes *in vivo* (Xiong et al., 2025; Wen et al., 2025). For example, DNA-based hydrogels—with their sequence programmability and dynamic adaptability—can balance structural support and biological function, providing a novel platform for engineering intelligent scaffolds for musculoskeletal regeneration (Shi et al., 2025). At the same time, next-generation bioprinters need to be capable of simultaneously and precisely depositing multiple cell types, growth factors and even artificially designed signal circuits, to recapitulate the inherent heterogeneity and complex signaling networks of native musculoskeletal tissues. The second key direction is to promote the in-depth integration of organ-on-a-chip technology with organoid and bioprinting technologies. By constructing perfusable musculoskeletal system microphysiological models that can apply precise mechanical or electrical stimuli, researchers can more faithfully mimic the *in vivo* microenvironment, enabling these models to be used for drug screening, disease mechanism research and verification of personalized treatment plans (Wen et al., 2025). This will greatly make up for the deficiencies of traditional two-dimensional cell culture and static three-dimensional organoid/bioprinted models. The third direction is to expand the application boundary of AI, moving beyond traditional data analysis toward dynamic simulation and virtual clinical trials. In the future, AI will be able to integrate multi-scale data (from molecular to tissue levels) to dynamically simulate the entire process of musculoskeletal regeneration—from cell recruitment and differentiation to tissue maturation and functional integration—thus predicting and optimizing regenerative strategies in the silicon-based virtual world (He et al., 2025b). For example, multimodal deep learning radiomics models have been successfully used to effectively predict the prognosis of patients after knee joint surgery, demonstrating the great potential of AI in clinical decision support for musculoskeletal disorders (He et al., 2025b). Furthermore, AI-driven virtual clinical trials are expected to significantly reduce the cost and cycle of regenerative therapy development, accelerating the advent of safe and effective novel therapies. The synergistic advancement of these directions ultimately aims to realize efficient, on-demand and fully personalized musculoskeletal regenerative therapy for patients with musculoskeletal injuries and degenerative diseases.

## 7 Tabular summaries of latest research (2020–2025)

### 7.1 Conclusion

The paradigm shift from static, homogenized regenerative strategies to dynamic, precise and personalized approaches marks a new era for musculoskeletal regenerative medicine. The in-depth integration of organoids, 3D/4D bioprinting, single-cell omics and AI is not a simple superposition of technologies, but the construction of a complete closed loop from the deciphering of basic regenerative mechanisms to the fabrication and optimization of clinical tissue products. Organoids, as physiologically relevant microenvironment models, provide an unprecedented window for understanding the molecular and cellular mechanisms of musculoskeletal tissue development and regeneration; 3D/4D bioprinting transforms this mechanistic understanding into functional biological substitutes with complex structures and dynamic response capabilities; single-cell omics technologies delve into the level of cellular heterogeneity and molecular regulatory networks, revealing key regenerative mechanisms that cannot be captured by traditional bulk analysis; AI, as the core engine of this integrated paradigm, efficiently integrates and deciphers massive multi-dimensional data, optimizes bioprinting parameters, predicts tissue functional performance, and assists clinical decision-making, greatly accelerating the translation process from “scientific discovery” to “clinical application”.

From an expert perspective, the core value of this multitechnological integration trend lies in its systematicness. It breaks the relatively isolated situation of each link in traditional research (mechanism research → model construction → clinical translation), and forms a highly efficient research system with mutual feedback and iterative optimization. For example, specific cell subsets or key signaling pathways discovered via single-cell omics can guide the optimized culture of musculoskeletal organoids or serve as key components of bioinks for bioprinting; the omics data generated by bioprinted tissue models can in turn be fed back to AI models for learning and optimization, further improving the design of tissue engineering strategies. This closed-loop workflow drastically improves the precision and efficiency of musculoskeletal regeneration research.

However, while embracing this promising future, we must carefully balance the viewpoint differences brought about by the different development stages of various technologies. For example, there are still many unresolved challenges and academic discussions regarding the degree of “physiological relevance” of musculoskeletal organoids, the long-term functional performance and biosafety of bioprinted tissue products, the complexity and interpretation of single-cell omics data, and the “black box” problem and clinical validation of AI models. The key to future development lies in in-depth interdisciplinary cooperation: materials scientists, biologists, clinicians and data scientists must work closely together to jointly formulate standardized validation systems for engineered musculoskeletal tissues, solve critical engineering challenges from laboratory-scale fabrication to large-scale production (e.g., bioreactor-based organoid maturation, vascularization of engineered tissues), and actively communicate with regulatory agencies to establish a clear and feasible approval path for these innovative regenerative products.

Despite the need to overcome significant challenges—such as technological integration complexity, high manufacturing costs and imperfect regulatory frameworks—the huge potential demonstrated by multitechnological integration in musculoskeletal regeneration is unquestionable. This integration is not only an upgrade of technical tools but also a fundamental innovation in research thinking and therapeutic concepts for musculoskeletal disorders. Through continuous technological iteration and cross-field collaboration, this integrated paradigm is expected to ultimately overcome the clinical treatment challenges of major musculoskeletal defects and degenerative diseases, and bring truly personalized and functional regenerative repair solutions to patients worldwide. The detailed methodological dissection, explicit *in vitro*/*in vivo* application spectrum and standardized tabular summaries provided in this review further enhance the rigor and translational value of this multitechnological integration paradigm, and provide a comprehensive and practical guide for researchers and clinicians in the field of musculoskeletal regeneration.
